# Adverse reactions to metal debris occur with all types of hip replacement not just metal-on-metal hips: a retrospective observational study of 3340 revisions for adverse reactions to metal debris from the National Joint Registry for England, Wales, Northern Ireland and the Isle of Man

**DOI:** 10.1186/s12891-016-1329-8

**Published:** 2016-12-13

**Authors:** Gulraj S. Matharu, Hemant G. Pandit, David W. Murray, Andrew Judge

**Affiliations:** 1 Nuffield Department of Orthopaedics, Rheumatology and Musculoskeletal Sciences, University of Oxford, Nuffield Orthopaedic Centre, Oxford, OX3 7LD UK; 2 Leeds Institute of Rheumatic and Musculoskeletal Medicine (LIRMM), Chapel Allerton Hospital, Chapeltown Road, Leeds, LS7 4SA UK; 3 MRC Lifecourse Epidemiology Unit, Southampton General Hospital, University of Southampton, Southampton, SO16 6YD UK

**Keywords:** Adverse reactions to metal debris, Hip replacement, Failure, Metal-on-metal, Non-metal-on-metal, Revision surgery

## Abstract

**Background:**

Adverse reactions to metal debris (ARMD) have resulted in the high short-term failure rates observed with metal-on-metal hip replacements. ARMD has recently been reported in non-metal-on-metal total hip replacements (non-MoM THRs) in a number of small cohort studies. However the true magnitude of this complication in non-MoM THRs remains unknown. We used a nationwide database to determine the risk of ARMD revision in all non-MoM THRs, and compared patient and surgical factors associated with ARMD revision between non-MoM and MoM hips.

**Methods:**

We performed a retrospective observational study using data from the National Joint Registry for England, Wales, Northern Ireland and the Isle of Man. All primary hip replacements undergoing revision surgery for ARMD were included (*n* = 3,340). ARMD revision risk in non-MoM THRs was compared between different commonly implanted bearing surfaces and femoral head sizes (Chi-squared test). Differences in patient and surgical factors between non-MoM hips and MoM hips revised for ARMD were also analysed (Chi-squared test and unpaired *t*-test).

**Results:**

Of all ARMD revisions, 7.5% (*n* = 249) had non-MoM bearing surfaces. The relative risk of ARMD revision was 2.35 times (95% CI 1.76–3.11) higher in ceramic-on-ceramic bearings compared with hard-on-soft bearings (0.055 vs. 0.024%; *p* < 0.001), and 2.80 times (95% CI 1.74–4.36) higher in 36 mm metal-on-polyethylene bearings compared to 28 mm and 32 mm metal-on-polyethylene bearings (0.058 vs. 0.021%; *p* < 0.001). ARMD revisions were performed earlier in non-MoM hips compared to MoM hips (mean 3.6-years vs. 5.6-years; *p* < 0.0001). Non-MoM hips had more abnormal findings at revision (63.1 vs. 35.7%; *p* < 0.001), and more intra-operative adverse events (6.4 vs. 1.6%; *p* < 0.001) compared to MoM hips.

**Conclusions:**

Although the overall risk of ARMD revision surgery in non-MoM THRs appears low, this risk is increasing, and is significantly higher in ceramic-on-ceramic THRs and 36 mm metal-on-polyethylene THRs. ARMD may therefore represent a significant clinical problem in non-MoM THRs.

## Background

Total hip replacement (THR) is the most successful surgical procedure for treating patients with hip arthritis [1]. 332,000 THRs are performed annually in the United States [2] with numbers expected to increase rapidly [3, 4]. Revision surgery for failed THR remains a significant problem, especially in young patients with high activity levels, with the future revision burden also expected to substantially increase [3–6].

Modifications were made to traditional THRs with metal-on-polyethylene bearing surfaces in an attempt to improve implant longevity and patient function. These included newer bearing surfaces with larger femoral head sizes, which aimed to reduce bearing wear and dislocation risk, whilst increasing hip movement. Furthermore, modular implants were an attractive concept to surgeons as they provided more flexibility in terms of helping to restore patient anatomy and optimising hip biomechanics [7]. As a result large-diameter metal-on-metal (MoM) bearing surfaces became popular with approximately 1.5 million of these designs implanted worldwide in young and active patients. However, MoM hips experienced high short-term failure rates [8, 9] with many revisions performed for adverse reactions to metal debris (ARMD) [10, 11]. The aetiology of ARMD remains incompletely understood. Initially excessive bearing wear was considered to be responsible, but more recently wear and corrosion at modular THR junctions has been implicated [12–14]. ARMD lesions are often invasive and destructive [10, 11], with poor outcomes reported following revision surgery [15]. Worldwide regulatory authorities therefore recommend regular follow-up for MoM hip patients [16, 17].

The three main bearing surfaces currently used in THR are metal-on-polyethylene, ceramic-on-ceramic, and ceramic-on-polyethylene [18, 19]. Recently ARMD requiring revision surgery has been observed in non-MoM THRs [20–24]. Small studies have reported between 7 and 27 ARMD revisions in non-MoM THRs, which mainly occurred in newer implant designs with large femoral heads [20–24]. One study estimated that 0.25% of consecutive non-MoM THRs (12 of 4813) implanted at their centre subsequently required revision for ARMD [23]. However, the true risk of revision surgery for ARMD in non-MoM THRs remains unknown.

The National Joint Registry (NJR) for England, Wales, Northern Ireland and the Isle of Man was established in 2003 to identify poorly performing implants early, and represents the world’s largest arthroplasty registry [18]. We assessed all hip replacements undergoing revision surgery for ARMD recorded in the NJR. The study aims were to: (1) determine the risk of ARMD revision surgery in all non-MoM hip replacements, and (2) compare patient and surgical factors associated with ARMD revision between non-MoM hip replacements and MoM hip replacements.

## Methods

We performed a retrospective observational study using data from the NJR for England, Wales, Northern Ireland and the Isle of Man. The NJR contains details of all primary and revision hip replacement procedures performed since April 2003. The present study was based on a subgroup of 3,433 hip replacements known to have required revision surgery for ARMD up until the 18^th^ November 2015. At the time this dataset was acquired the NJR had recorded a total of 889,340 primary hip replacement procedures.

Numerous terms have been used to describe abnormal destructive reactions related to MoM hip replacements that require revision surgery. These include ARMD [11, 14], pseudotumour [10], aseptic lymphocytic vasculitis-associated lesions [25], and adverse local tissue reaction [26]. These terms are often used interchangeably to describe the same process. In June 2008, the NJR first introduced the term ARMD for surgeons to select as an indication for revision surgery, given ARMD is considered the most inclusive term for these abnormal reactions [11, 14]. The present study includes all primary hip replacements revised for ARMD between 1st June 2008 and 18th November 2015, which have been recorded in the NJR.

By using unique patient identifiers all 3,433 revision procedures for ARMD could be linked to the primary hip replacement procedure. For both the primary and revision procedures the NJR collects data on patient demographics (age, gender, body mass index, American Society of Anesthesiologists (ASA) grade, indication for surgery, venous thromboembolism prophylaxis), surgery performed (surgeon grade, surgical approach, details of components implanted including the bearing surface, component size, and implant fixation), and intra-operative adverse surgical events (calcar crack; pelvic and/or femoral shaft penetration; trochanteric and/or femoral shaft fracture; other), which were all available for analysis in the present study. In addition, NJR data on revision procedures provided detailed information on intra-operative findings, including reason(s) for revision surgery. After linking primary and revision procedures the bearing surface implanted at the primary surgery could not be clearly identified in 93 cases. These were excluded, leaving 3,340 hip revisions performed for ARMD in the final study cohort for analysis. Conventional methods for describing bearing surfaces were used, for example metal-on-polyethylene represents a metal femoral head articulating with a polyethylene liner or socket.

### Statistical analysis

The cohort was divided into two groups based on whether the primary hip replacement bearing surface was non-MoM or MoM. The MoM group included both stemmed THRs and hip resurfacings. Subsequent analyses were separated into two distinct parts to reflect the study aims.

#### Risk of ARMD revision surgery in non-MoM hip replacements

To calculate the risk of ARMD revision surgery, observational data for all hip replacements revised for ARMD as recorded in the NJR were available as the numerator. Complete clinical data on all hip replacements not undergoing revision surgery were not available. For the denominator, data from the NJR were provided on the total number of primary hip replacements performed as a whole, and for different bearing surfaces and femoral head sizes. Hence it was possible to calculate the risk of ARMD revision surgery for the whole NJR population, and for each bearing surface and femoral head size subgroup. The relative risk of ARMD revision between different non-MoM bearing surfaces and femoral head sizes were compared using the Chi-squared test with Yates’ correction.

#### Comparison of patient and surgical factors associated with ARMD revision between non-MoM and MoM hip replacements

Patients were selected for this study based on their final outcome, i.e., revision of a primary hip replacement for ARMD. Patient and surgical factors (for both the primary hip replacement and the revision surgery for ARMD) were subsequently compared between non-MoM hip replacements (cases) and MoM hip replacements (controls). Data from all numerical co-variates were normally distributed and compared using unpaired *t*-tests, with categorical data assessed using either the Chi-squared test with Yates’ correction or Fisher’s exact test. Time to ARMD revision surgery was also assessed using the Kaplan-Meier method, with a univariate Cox proportional hazards model used to compare time to revision by type of primary bearing surface. As the analysis only included observational data on hip replacements revised for ARMD, the different primary bearing surface groups in this Cox model all reached a survival probability of zero. Multivariable logistic regression modeling was used to assess the effect of patient demographics (age, gender, ASA grade, indication for primary surgery, time to revision surgery) on the binary outcome variable (whether an ARMD revision was performed in a non-MoM or MoM hip replacement). Surgical factors (such as surgical approach, component size, and implant fixation) were not included in the logistic regression models given these factors are almost completely determined by the initial decision to perform either a primary non-MoM or MoM hip replacement, hence these surgical factors are part of the causal pathway [9]. When assessing patient demographics in the logistic regression models, linearity of continuous predictors was assessed using fractional polynomials with data grouped if affects were non-linear. All analyses were performed using Stata Version 13.1 (Lakeway Drive, Texas, USA) with 95% confidence intervals (CIs) provided for all estimates.

## Results

### Risk of ARMD revision surgery

**Fig. 1:**
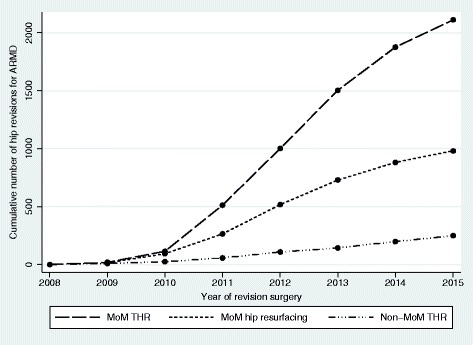
Cumulative number of hip revision procedures performed for ARMD between 2008 and 2015 stratified by the type of primary hip replacement. ARMD = adverse reactions to metal debris; MoM = metal-on-metal; THR = total hip replacement

Of 3,340 primary hip replacements undergoing revision surgery for ARMD, 249 (7.5%) had non-MoM bearing surfaces with the remaining 3,091 (92.5%) hips having MoM bearings (Fig. 1).

During the study period a total of 873,188 primary hip replacements were recorded in the NJR where the primary bearing surface could be correctly identified. The risk of ARMD revision surgery in all implanted primary hip replacements was 0.38% (3,340/873,188; 95% CI 0.37–0.40%). The risk of ARMD revision surgery in all non-MoM hip replacements recorded in the NJR was 0.032% (249/789,397; 95% CI 0.028–0.036%) compared to 3.7% (3,091/83,791; 95% CI 3.6–3.8%) in MoM hip replacements (*p* < 0.001).

When non-MoM hip replacements were subdivided by bearing surface, the risk of ARMD revision surgery by primary bearing surface was: metal-on-polyethylene 0.024% (125/526,951; 95% CI 0.020–0.028%), ceramic-on-ceramic 0.055% (75/135,267; 95% CI 0.044–0.070%), ceramic-on-polyethylene 0.023% (29/124,656; 95% CI 0.016–0.033%), ceramic-on-metal 0.69% (16/2,320; 95% CI 0.39–1.12%), and metal-on-ceramic 1.97% (4/203; 95% CI 0.54–4.97%).

Although ceramic-on-metal and metal-on-ceramic bearings had the highest risk of ARMD revision for all non-MoM bearings, they were implanted in small numbers and are no longer used. When these two non-MoM bearing surfaces were excluded, the risk of ARMD revision in non-MoM hip replacements was dependent on bearing surface and femoral head size. The relative risk of ARMD revision was 2.35 times (95% CI 1.76–3.11) higher in hard-on-hard (ceramic-on-ceramic) compared with hard-on-soft bearing surfaces (metal-on-polyethylene and ceramic-on-polyethylene) (*p* < 0.001). The relative risk of ARMD revision was 2.80 times (95% CI 1.74–4.36) higher in 36 mm metal-on-polyethylene bearings compared to 28 mm and 32 mm (28/32 mm) metal-on-polyethylene bearings (*p* < 0.001; Table 1). The risk of ARMD revision was not influenced by femoral head size in both ceramic-on-ceramic and ceramic-on-polyethylene bearing surfaces (Table 1). However, the risk of ARMD revision in both 28/32 mm (0.057%) and 36 mm (0.052%) ceramic-on-ceramic bearings was similar to 36 mm metal-on-polyethylene bearings (0.058%).

**Table 1:** Risk of ARMD revision surgery for the three most commonly implanted primary hip replacement bearing surfaces by femoral head size

Hip bearing surface	Femoral head size (mm)	Number of hips implanted	Number of hips revised for ARMD	Risk of ARMD revision surgery (%) (95% CI)	Relative risk (95% CI) of ARMD revision with 36 mm head size (vs. 28/32 mm)	*p*-value (36 mm vs. 28/32 mm for same bearing surface)
MoP	28 & 32	407,412	85	0.021 (0.017–0.026)	2.80 (1.74–4.36)	<0.001
MoP	36	46,258	27	0.058 (0.038–0.085)		
CoC	28 & 32	61,937	35	0.057 (0.039–0.079)	0.92 (0.56–1.51)	0.725
CoC	36	67,373	35	0.052 (0.036–0.072)		
CoP	28 & 32	96,286	24	0.025 (0.016–0.037)	0.82 (0.24–2.19)	0.683
CoP	36	24,513	5	0.020 (0.007–0.048)		

*ARMD* Adverse reactions to metal debris, *CoC* Ceramic-on-ceramic, *CoP* Ceramic-on-polyethylene, *MoP* Metal-on-polyethylene, *CI* Confidence interval

### Differences at primary surgery between non-MoM and MoM hips subsequently revised for ARMD (Table 2)

**Table 2:** Patient and surgical factors relating to the primary hip replacement procedure in all hips subsequently revised for ARMD

Co-variate	All ARMD revisions (*n* = 3340) (100%)	ARMD revisions in MoM hips (*n* = 3091) (92.5%)	ARMD revisions in non-MoM hips (*n* = 249) (7.5%)	*p*-value	Odds ratio (95% CI)
Gender					
Female vs. *male*	1,967 (58.89)	1,810 (58.56)	157 (63.05)	0.165	0.87 (0.66–1.14)
Age (in years)^a^					
Mean (SD)	58.8 (10.0)	58.4 (9.8)	63.8 (11.0)	<0.0001	
< 50	557 (16.68)	534 (17.28)	23 (9.24)	<0.001	Ref
50–59	1,113 (33.32)	1,061 (34.33)	52 (20.88)		1.12 (0.67–1.87)
60–69	1,227 (36.74)	1,134 (36.69)	93 (37.35)		1.81 (1.11–2.93)
≥ 70	443 (13.26)	362 (11.71)	81 (32.53)		4.72 (2.86–7.78)
BMI (in kg/m^2^)^b^					
Mean (SD)	28.3 (4.8)	28.3 (4.9)	28.3 (4.4)	0.978	-
Primary hip design					
THR	2,359 (70.63)	2110 (68.3)	249 (100)	NA	-
HR	981 (29.37)	981 (31.7)	0 (0)		
Bearing surface					
MoM	3,091 (92.54)	3091 (100)	0 (0)	NA	-
MoP	125 (3.74)	0 (0)	125 (50.20)		
CoC	75 (2.25)	0 (0)	75 (30.12)		
CoP	29 (0.87)	0 (0)	29 (11.65)		
CoM	16 (0.48)	0 (0)	16 (6.43)		
MoC	4 (0.12)	0 (0)	4 (1.61)		
Femoral head size (mm)					
Mean (SD)	43.0 (6.4)	43.9 (5.7)	32.1 (4.0)	<0.0001	-
Range	22.25–60	28–60	22.25–44		
Median	44	46	32		
Inter-quartile range	36–48	38–48	28–36		
≤ 28	143 (4.28)	44 (1.42)	99 (39.76)	<0.001	
32	55 (1.65)	0 (0)	55 (22.09)		
36–42	1,197 (35.84)	1,103 (35.68)	94 (37.75)		
44–48	1,373 (41.11)	1,372 (44.39)	1 (0.40)		
50–52	449 (13.44)	449 (14.53)	0 (0)		
≥ 54	123 (3.68)	123 (3.98)	0 (0)		
Cup fixation^b^					
Uncemented	3,285 (98.73)	3,078 (99.96)	207 (83.46)	<0.001	-
Cemented	42 (1.26)	1 (0.04)	41 (16.53)		
Stem fixation (THR only)^b^					
Uncemented	2,090 (91.15)	1,922 (93.85)	168 (68.57)	<0.001	-
Cemented	203 (8.85)	126 (6.15)	77 (31.43)		
ASA grade					
1	1,228 (36.77)	1,169 (37.82)	59 (23.69)	<0.001	Ref
2	1,948 (58.32)	1,776 (57.46)	172 (69.08)		1.61 (1.18–2.20)
3 or above	164 (4.91)	14 (4.72)	18 (7.23)		1.68 (0.95–2.97)
Indication for surgery					
Primary OA vs. *other*	3,139 (93.98)	2,906 (94.01)	233 (93.57)	0.779	0.73 (0.42–1.26)
Surgeon grade					
Consultant vs. *other*	2,909 (87.10)	2,699 (87.32)	210 (84.34)	0.177	-
Complex primary	174 (5.21)	161 (5.21)	13 (5.22)	0.993	-
Surgical approach^b^					
Posterior vs. *other*	2,241 (69.04)	2,109 (70.23)	132 (54.32)	<0.001	-
VTE - chemical					
LMWH (+/-other)	1,673 (50.09)	1,524 (49.30)	149 (59.84)	<0.001	-
Aspirin only	419 (12.54)	401 (12.97)	18 (7.23)		
Other	197 (5.90)	150 (4.85)	47 (18.88)		
None	1,051 (31.47)	1,016 (32.87)	35 (14.06)		
VTE - mechanical					
Any vs. *none*	2,919 (87.40)	2,690 (87.03)	229 (91.97)	0.024	-
Adverse surgical events^c^	42 (1.83)	35 (1.68)	7 (3.30)	0.102	-

*ARMD* Adverse reactions to metal debris, *ASA* American Society of Anesthesiologists, *BMI* Body mass index, *CoC* Ceramic-on-ceramic, *CoM* Ceramic-on-metal, *CoP* Ceramic-on-polyethylene, *CI* Confidence interval, *HR* Hip resurfacing, *LMWH* Low molecular weight heparin, *MoC* Metal-on-ceramic, *MoM* Metal-on-metal, *MoP* Metal-on-polyethylene, *NA* Not applicable, *OA* Osteoarthritis, *Ref* Reference group, *SD* Standard deviation, *THR* Total hip replacement, *VTE* Venous thrombo-embolism

Values in brackets are percentages unless otherwise indicated. Statistically significant differences between the two groups (*p* < 0.05) have been highlighted in bold text

^a^Affect of age at primary surgery on outcome was non-linear so variable was grouped

^b^Missing data for stated number of hips: BMI (*n* = 2246); surgical approach (*n* = 94); cup fixation (*n* = 13); THR stem fixation (*n* = 66)

^c^Intra-operative adverse events included: calcar crack; pelvic and/or femoral shaft penetration; trochanteric and/or femoral shaft fracture; other

-Co-variate was not included in multivariable logistic regression model as either it was a surgical factor that is part of the causal pathway, or there was significant missing data

Non-MoM hip patients were significantly older (*p* < 0.0001) with higher ASA grades (*p* < 0.001) at primary surgery. Significantly larger femoral head sizes were implanted in MoM hips at primary surgery (femoral head sizes ≥36 mm implanted in 98.6% of MoM hips vs. 38.2% of non-MoM hips; *p* < 0.001).

### Differences at revision surgery between non-MoM and MoM hips revised for ARMD (Table 3)

**Table 3:** Patient and surgical factors relating to the revision hip replacement procedure performed for ARMD

Co-variate	All ARMD revisions (*n* = 3340) (100%)	ARMD revisions in MoM hips (*n* = 3091) (92.5%)	ARMD revisions in non-MoM hips (*n* = 249) (7.5%)	*p*-value	Odds ratio (95% CI)
Gender					
Female vs. *male*	1,967 (58.89)	1,810 (58.56)	157 (63.05)	0.165	0.81 (0.62–1.07)
Age (in years)^a^					
Mean (SD)	64.2 (10.1)	64.0 (10.0)	67.5 (10.9)	<0.0001	
< 50	271 (8.11)	255 (8.25)	16 (6.43)	<0.001	Ref
50–59	712 (21.32)	672 (21.74)	40 (16.06)		0.94 (0.51–1.72)
60–69	1,292 (38.68)	1,214 (39.28)	78 (31.33)		1.17 (0.66–2.06)
≥ 70	1,065 (31.89)	950 (30.73)	115 (46.18)		2.34 (1.33–4.12)
BMI (in kg/m^2^)^b^					
Mean (SD)	29.0 (5.2)	29.0 (5.2)	28.5 (5.3)	0.285	-
Bilateral ARMD revisions	282 (8.44)	281 (9.09)	1 (0.40)	<0.001	-
Time from primary to revision (in years)^a^					
Mean (SD)	5.4 (2.1)	5.6 (2.0)	3.6 (2.9)	<0.0001	
≤ 5	1,387 (41.53)	1,209 (39.11)	178 (71.49)	<0.001	Ref
> 5	1,953 (58.47)	1,882(60.89)	71 (28.51)		0.23 (0.17–0.30)
ASA grade					
1	609 (18.23)	569 (18.41)	40 (16.06)	0.012	Ref
2	2,389 (71.53)	2,219 (71.79)	170 (68.27)		1.01 (0.70–1.47)
3 or above	342 (10.24)	303 (9.80)	39 (15.66)		1.70 (1.04–2.78)
VTE - chemical					
LMWH (+/-other)	1,798 (53.83)	1,644 (53.19)	154 (61.85)	0.002	-
Aspirin only	92 (2.75)	80 (2.59)	12 (4.82)		
Other	1,155 (34.58)	1,093 (35.36)	62 (24.90)		
None	295 (8.83)	274 (8.86)	21 (8.43)		
VTE - mechanical					
Any vs. *none*	3,189 (95.48)	2,957 (95.66)	232 (93.17)	0.069	-
Surgeon grade					
Consultant vs. *other*	3,201 (95.84)	2,963 (95.86)	238 (95.58)	0.833	-
Surgical approach^b^					
Posterior vs. *other*	2,691 (81.87)	2,516 (82.55)	175 (73.22)	<0.001	-
Revision type					
Single stage	3,266 (97.78)	3,034 (98.16)	232 (93.17)	<0.001	-
Staged (2 or more)	71 (2.13)	54 (1.75)	17 (6.83)		
Excision arthroplasty	3 (0.09)	3 (0.10)	0 (0)		
Revision indications/intra-operative findings^c^					
1 indication	2,080 (62.28)	1,988 (64.32)	92 (36.95)	<0.001	-
2 to 6 indications	1,260 (37.72)	1,103 (35.68)	157 (63.05)		
ARMD	3340 (100)	3091 (100)	249 (100)	NA	-
Pain	665 (19.91)	618 (19.99)	47 (18.88)	0.671	-
Aseptic loosening	338 (10.12)	278 (8.99)	60 (24.10)	<0.001	-
Osteolysis	255 (7.63)	221 (7.15)	34 (13.65)	<0.001	-
Implant malalignment	118 (3.53)	96 (3.11)	22 (8.84)	<0.001	-
Other indications/findings	101 (3.02)	98 (3.17)	3 (1.20)	0.084	-
Acetabular component wear	83 (2.49)	46 (1.49)	37 (14.86)	<0.001	-
Dislocation/subluxation	67 (2.01)	41 (1.33)	26 (10.44)	<0.001	-
Fracture	66 (1.98)	54 (1.75)	12 (4.82)	0.003	-
Infection	43 (1.29)	33 (1.07)	10 (4.02)	0.001	-
Liner dissociation	25 (0.75)	10 (0.32)	15 (6.02)	<0.001	-
Implant fracture	18 (0.54)	7 (0.23)	11 (4.42)	<0.001	-
Incorrect implant size	17 (0.51)	11 (0.36)	6 (2.41)	0.001	-
Revision procedure^b,d^					
All components revised	1,267 (38.79)	1,202 (39.62)	65 (28.02)	<0.001	-
Cup (+/- head/liner/taper)	1,426 (43.66)	1,350 (44.50)	76 (32.76)		
Stem (+/- head/liner/taper)	144 (4.41)	107 (3.53)	37 (15.95)		
Head/ liner/taper revision only	422 (12.92)	370 (12.20)	52 (22.41)		
Femoral head size (mm)^b,d^					
Mean (SD)	34.1 (3.3)	34.1 (3.2)	33.2 (3.7)	0.0003	-
Range	22.25–48	22.25–48	22.25–40		
Median	36	36	36		
Inter-quartile range	32–36	32–36	32–36		
≤ 32	1,180 (37.94)	1,073 (37.09)	107 (49.31)	0.001	
36	1,783 (57.33)	1,683 (58.17)	100 (46.08)		
> 36	147 (4.73)	137 (4.74)	10 (4.61)		
Bearing surface^b,d^					
CoP	1,479 (47.6)	1,394 (48.4)	85 (38.1)	<0.001	-
CoC	885 (28.5)	841 (29.2)	44 (19.7)		
MoP	736 (23.7)	642 (22.3)	94 (42.2)		
CoM	3 (0.10)	3 (0.10)	0 (0)		
MoM	2 (0.06)	2 (0.07)	0 (0)		
MoC	1 (0.03)	1 (0.03)	0 (0)		
Cup fixation^d^					
Uncemented	2,355 (87.45)	2,239 (87.74)	116 (82.27)	0.057	-
Cemented	338 (12.55)	313 (12.26)	25 (17.73)		
Stem fixation^d^					
Uncemented	867 (61.45)	819 (62.57)	48 (47.06)	0.002	-
Cemented	544 (38.55)	490 (37.43)	54 (52.94)		
Bone graft (femoral)^d^	107 (3.20)	99 (3.20)	8 (3.21)	0.993	-
Bone graft (acetabular)^d^	650 (19.46)	611 (19.77)	39 (15.66)	0.116	-
Adverse surgical events^d,e^	64 (1.92)	48 (1.55)	16 (6.43)	<0.001	-

*ARMD* Adverse reactions to metal debris, *ASA* American Society of Anesthesiologists, *BMI* Body mass index, *CoC* Ceramic-on-ceramic, *CoM* Ceramic-on-metal, *CoP* Ceramic-on-polyethylene, *CI* Confidence interval, *HR* Hip resurfacing, *LMWH* Low molecular weight heparin, *MoC* Metal-on-ceramic, *MoM* Metal-on-metal, *MoP* Metal-on-polyethylene, *NA* Not applicable; *Ref* Reference group, *SD* Standard deviation, *THR* Total hip replacement, *VTE* Venous thrombo-embolism

Values in brackets are percentages unless otherwise indicated. Statistically significant differences between the two groups (*p* < 0.05) have been highlighted in bold text

^a^Affect of age at revision surgery on outcome, and affect of time to revision surgery on outcome were non-linear so variables were grouped

^b^Missing data for stated number of hips: BMI (*n* = 886); surgical approach (*n* = 53); revision procedure (*n* = 7); femoral head size (*n* = 156); bearing surface (*n* = 160)

^c^Intra-operative revision findings refer to a problem with one or both hip components (loosening, fracture etc.)

^d^All details about the specific revision surgical procedures performed are provided for the 3266 hips (97.8% of the cohort) undergoing single stage revisions. Therefore details about 74 hip revisions undergoing either 2 or more stages, or excision arthroplasty procedures have not been included

^e^Intra-operative adverse events included: calcar crack; pelvic and/or femoral shaft penetration; trochanteric and/or femoral shaft fracture; other

- Co-variate was not included in multivariable logistic regression model as either it was a surgical factor that is part of the causal pathway, or there was significant missing data

**Fig. 2:**
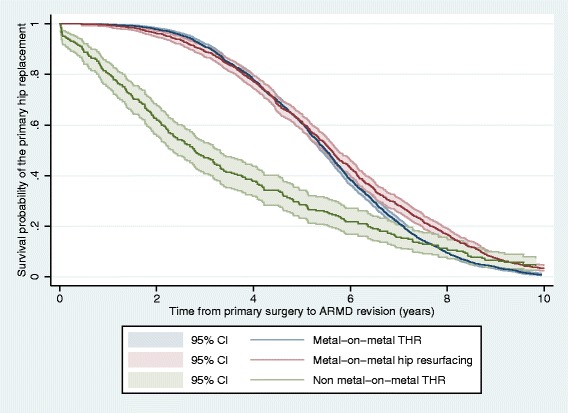
Kaplan Meier ARMD revision rate stratified by the type of primary hip replacement. ARMD = adverse reactions to metal debris; CI = confidence intervals; THR = total hip replacement. The study population all underwent revision of their primary hip replacement implant for ARMD, therefore in the illustrated Kaplan Meier plot all three subgroups ultimately reach a survival probability of zero. This is because complete clinical data for all primary hip replacements not undergoing revision were not available for analysis in this study

Revision for ARMD was performed significantly earlier in non-MoM hips compared to MoM hips (mean time from primary to revision surgery 3.6 vs. 5.6 years; *p* < 0.0001; Fig. 2). Non-MoM hips were significantly more likely to undergo staged revision procedures than MoM hips (6.8 vs. 1.8%; *p* < 0.001), and were significantly more likely to have other abnormalities at revision (63.1 vs. 35.7%; *p* < 0.001), including aseptic component loosening (*p* < 0.001), osteolysis (*p* < 0.001), implant malalignment (*p* < 0.001), dislocation/subluxation (*p* < 0.001), fracture (*p* = 0.003), and infection (*p* = 0.001). Significantly more intra-operative adverse events (including pelvic and/or femoral shaft penetration/fractures) occurred in non-MoM hips revised for ARMD compared to MoM hips (6.4 vs. 1.6%; *p* < 0.001).

## Discussion

Prior to this study it was not known whether ARMD associated with non-MoM hip replacements represented a significant clinical problem. Analysis of the world’s largest arthroplasty database has demonstrated that although the risk of revision surgery for ARMD in non-MoM THRs was low, the risk is increasing with one-in-thirteen ARMD revisions performed in non-MoM hip replacements. Furthermore, the risk of ARMD revision was significantly higher in ceramic-on-ceramic THRs and 36 mm metal-on-polyethylene THRs.

### Overall risk of revision surgery for ARMD

The observation that 7.5% of all ARMD revisions were performed in non-MoM THRs is high, especially given the small number of cases reported worldwide [20–24]. Coupled with the annual increasing trend of ARMD revisions in non-MoM THRs these observations are concerning. As only limited reports have been published [20–24] there is currently a relative lack of awareness amongst clinicians that ARMD associated with non-MoM hips represents a significant problem. Therefore our findings are likely to be influenced by surveillance bias and the true ARMD revision risk is potentially underestimated. The number of ARMD revisions performed in MoM hips were also heavily influenced by surveillance bias given revisions increased considerably between 2010 and 2013, which reflects widespread recognition of this problem and the implementation of regular patient follow-up [16, 17]. If closer surveillance is deemed necessary for non-MoM THRs [7], the risk of ARMD revision is expected to increase at a greater rate than presently.

The risk of ARMD revision in the three commonest non-MoM bearing surfaces appears low (0.023–0.055%) as this entity is not well recognised and the overall number of THRs implanted is very large. However, the risk of ARMD revision in MoM hips in 2015 (3.7%) has increased 25-fold compared to that reported in 2009 (0.15%), when little was known about ARMD in MoM hips [27]. Although we suspect the risk of ARMD revision in non-MoM hips will not reach levels observed in MoM hips, the risk may increase at a similar rate.

### ARMD risk by bearing surface and femoral head size

ARMD revisions were performed in all commonly implanted non-MoM bearing surfaces and all femoral head sizes. However, the risk of ARMD revision was 2.35 times higher in ceramic-on-ceramic bearings compared with hard-on-soft bearings, and 2.80 times higher in 36 mm metal-on-polyethylene bearings compared to smaller metal-on-polyethylene THRs.

Ceramic-on-ceramic THRs became popular for treating young and active patients with hip arthritis, especially since high failure rates associated with MoM bearings were recognised [18, 19]. Analysis of NJR data in 2012 observed low all-cause revision rates in large-diameter ceramic-on-ceramic bearings, therefore the continued use of ceramic-on-ceramic THRs with large femoral head sizes was recommended [8]. By contrast, with regard to the risk of ARMD revision our data demonstrates that ceramic-on-ceramic THRs have a significantly higher relative risk compared to metal-on-polyethylene and ceramic-on-polyethylene bearings. When choosing bearing surfaces for primary THR we recommend surgeons carefully consider the competing risks of all-cause versus ARMD revision, the potentially devastating complication of ARMD [10, 15], and acknowledge that ARMD revision rates in non-MoM THRs may be much higher than we have reported due to a lack of patient surveillance and incorrect surgeon reporting. However, our findings do not support the use of ceramic-on-ceramic THRs of any head size over hard-on-soft bearings if the risk of ARMD revision is to be minimised.

Metal-on-polyethylene remains the most commonly implanted THR bearing worldwide [18, 19]. Recently larger head sizes have been implanted to reduce dislocation risk and potentially reduce wear. Our observations suggest 36 mm metal-on-polyethylene bearings have a significantly increased risk of ARMD revision compared to smaller sizes, and an ARMD risk similar to ceramic-on-ceramic bearings. Therefore we recommend against using 36 mm or above metal-on-polyethylene THRs if smaller bearings can safely be implanted. The risk of ARMD revision was low in 28/32 mm and 36 mm ceramic-on-polyethylene THRs suggesting it could be safe to use 36 mm femoral heads with ceramic-on-polyethylene THRs. However, definitive conclusions cannot be drawn given much fewer 36 mm ceramic-on-polyethylene THRs were implanted compared to ceramic-on-ceramic and metal-on-polyethylene THRs.

### Mechanisms for findings

Although we have some understanding of the mechanisms underlying ARMD development in MoM hips [12–14], we currently do not understand why ARMD occurs in non-MoM THRs which is extremely concerning. Some implicate corrosion at modular implant junctions (femoral head-neck junction and femoral neck-stem junction) [20–24], with corrosion occurring due to articulating mixed alloys, such as metal femoral heads with titanium femoral necks [28]. Ceramic-on-ceramic bearings may develop ARMD because of high friction causing metal debris at the trunnion and/or other modular junctions. Large metal-on-polyethylene bearings may be increasingly prone to wear and corrosion at the femoral head-neck junction and/or other modular junctions because of increased transmitted torques from larger heads [8]. Further research is needed to establish why ARMD develops in non-MoM THRs.

### Primary surgery factors

Numerous differences existed between non-MoM and MoM hip patients at primary surgery. These relate to the inherent selection bias for undergoing each procedure [9] and are factors causally related to the primary procedure, rather than truly clinically significant differences in ARMD revision between different bearing surfaces.

### Revision surgery factors

ARMD revisions were performed significantly earlier in non-MoM THRs compared to MoM hips. This is concerning given MoM hips have high short-term failure rates [8, 9]. We can only speculate reasons for this difference. In addition to ARMD, non-MoM THRs had significantly more abnormal findings at revision compared to MoM hips. A number of these (aseptic loosening, osteolysis, implant malalignment) are readily identifiable on hip radiographs. The presence of such abnormalities at an early stage of investigation may have contributed towards earlier revision compared to MoM hips, with ARMD subsequently diagnosed at revision surgery. Another explanation relates to the disease process. It is possible that ARMD due to corrosion is more aggressive than ARMD developing from high bearing wear, with evidence in MoM hips supporting much higher failure rates in THRs compared to hip resurfacings even with identical bearing surfaces [14, 18]. However this requires further investigation, including implant retrieval and histopathological analysis.

Non-MoM THRs more commonly underwent staged revisions compared to MoM hips. As registries do not record histopathological data we can again only speculate an explanation for this observation. Given surgeons are presently less aware of ARMD associated with non-MoM THRs compared to MoM hips, and that the intra-operative appearances of ARMD can be similar to those seen with infection, it is possible that surgeons were more likely to elect to treat failing non-MoM THRs with staged revisions rather than in a single stage. Staged revisions in non-MoM THRs may also have been preferable given these hips had significantly more abnormal findings at revision which may have required major reconstruction.

The observation that non-MoM THRs have significantly more adverse events at revision surgery is also concerning. This may again relate to the increased number of abnormalities at revision in non-MoM hips making the surgery more complex. Furthermore, it is expected that removing a well-fixed corroded femoral component in non-MoM THRs is more likely to be associated with complications, such as fracture, compared with removing MoM hip resurfacings which conserve femoral bone. Revision of MoM hips for ARMD has resulted in generally poor short-term outcomes [15]. Given that non-MoM THRs had more abnormal findings and adverse events at revision surgery compared to MoM hips, it is hypothesised they may have poor short-term outcomes. However limited evidence is currently available [24], therefore future studies must establish outcomes following ARMD revision in non-MoM THRs.

### Strengths

Study strengths include the dataset coming from the worlds largest arthroplasty registry which uses linked data to ensure procedures performed at different institutions were captured, with almost complete compliance now reported [29]. Only small case series are presently available [20–24], therefore our study contributes significantly to the literature. Furthermore, by reporting on the whole population our study is not subject to sampling bias. Given the cohort size and that THR is so common worldwide with similar bearing surfaces and femoral head sizes implanted [3, 19], it is suspected our findings have good external validity and generalisability, though this requires formal validation.

### Limitations

Although our study is large, it is based on observational data therefore it is difficult to infer causality. However, we have provided explanations for our findings based on the literature and suggested important future research. The risk of ARMD revision in non-MoM hips reported here is likely to be an underestimate given surgeons may not have been aware of this problem with these bearings, and therefore incorrectly coded revisions using other indications, such as infection. It is also possible some ARMD revisions were performed but not recorded which would also underestimate the problem [30]. Surgeons may also have different thresholds for diagnosing ARMD at revision surgery, which may influence the study findings. It was not possible to confirm the diagnosis of ARMD histopathologically using registry data. Although this is an important limitation of the present study, we recommend future prospective studies based on non-registry cohorts report details of their histopathological analysis and confirm the diagnosis of ARMD. As ARMD can be difficult to distinguish from infection by the surgeon at the time of revision, it is also possible that given the lack of histopathological data some staged and non-staged ARMD revisions may actually have been for infection rather than ARMD. Therefore the risk of ARMD revision in non-MoM THRs may have been overestimated here. Finally, it was not possible to access data regarding the specific hip implant designs given this is considered sensitive information by the NJR and manufacturers. However we recognise the importance of analysing this given the significant changes recently made to THR designs.

## Conclusions

We observed a significant proportion (7.5%) of all ARMD revisions occur in non-MoM THRs. Although the overall risk of ARMD revision surgery in non-MoM THRs appears low, it is increasing, and is significantly higher in ceramic-on-ceramic THRs and in 36 mm metal-on-polyethylene THRs. Compared to MoM hips, non-MoM THR ARMD revisions were performed significantly earlier and had significantly more abnormalities and adverse events at revision surgery. ARMD associated with non-MoM THRs may represent a significant clinical problem that will become more apparent with time. For primary THR we recommend using hard-on-soft bearings with 28/32 mm femoral heads where possible, as this will minimise the clinical impact of ARMD in non-MoM hips. Further work is needed to establish whether ARMD development in non-MoM THRs is specific to certain implant designs.

## Abbreviations

ARMD: Adverse reactions to metal debris
ASA: American Society of Anesthesiologists
CI: confidence interval
MoM: metal-on-metal
NJR: National Joint Registry
non-MoM THR: non-metal-on-metal total hip replacement
THR: total hip replacement
